# Wireless Technology Recognition Based on RSSI Distribution at Sub-Nyquist Sampling Rate for Constrained Devices

**DOI:** 10.3390/s17092081

**Published:** 2017-09-12

**Authors:** Wei Liu, Merima Kulin, Tarik Kazaz, Adnan Shahid, Ingrid Moerman, Eli De Poorter

**Affiliations:** Ghent University-imec, IDLab, Department of Information Technology, B-9052 Gent, Belgium; merima.kulin@ugent.be (M.K.); Tarik.kazaz@ugent.be (T.K.); adnan.shahid@ugent.be (A.S.); Ingrid.moerman@ugent.be (I.M.); eli.depoorter@ugent.be (E.D.P.)

**Keywords:** technology recognition, RSSI, multi-modal distribution, experimental study, constrained devices, NGWN

## Abstract

Driven by the fast growth of wireless communication, the trend of sharing spectrum among heterogeneous technologies becomes increasingly dominant. Identifying concurrent technologies is an important step towards efficient spectrum sharing. However, due to the complexity of recognition algorithms and the strict condition of sampling speed, communication systems capable of recognizing signals other than their own type are extremely rare. This work proves that multi-model distribution of the received signal strength indicator (RSSI) is related to the signals’ modulation schemes and medium access mechanisms, and RSSI from different technologies may exhibit highly distinctive features. A distinction is made between technologies with a streaming or a non-streaming property, and appropriate feature spaces can be established either by deriving parameters such as packet duration from RSSI or directly using RSSI’s probability distribution. An experimental study shows that even RSSI acquired at a sub-Nyquist sampling rate is able to provide sufficient features to differentiate technologies such as Wi-Fi, Long Term Evolution (LTE), Digital Video Broadcasting-Terrestrial (DVB-T) and Bluetooth. The usage of the RSSI distribution-based feature space is illustrated via a sample algorithm. Experimental evaluation indicates that more than 92% accuracy is achieved with the appropriate configuration. As the analysis of RSSI distribution is straightforward and less demanding in terms of system requirements, we believe it is highly valuable for recognition of wideband technologies on constrained devices in the context of dynamic spectrum access.

## 1. Introduction

Due to the quick growth of wireless communications, radio spectrum bands are either already claimed by licensed users or heavily loaded by applications in the unlicensed bands. The shortage of spectrum resource has become a key limitation of wireless communications. One way to resolve the issue is to extend the spectrum towards a higher frequency band via new technologies, such as millimeter wave communication [1] assisted by massive MIMO [2]; another way is to improve the efficiency of the already allocated frequency bands by allowing dynamic spectrum access (DSA) [3].

DSA essentially means sharing spectrum among heterogeneous technologies, which can be achieved either “horizontally” [4] or “vertically” [5]. Vertical spectrum sharing refers to the opportunistic access of licensed bands without compromising the incumbents’ communication quality, while horizontal spectrum sharing refers to the access of unlicensed bands by multiple technologies with equal privileges. Several technologies (e.g., Wi-Fi, ZigBee, Bluetooth) are already sharing the Industrial Scientific and Medical (ISM) bands in the horizontal style. Attracted by the free spectrum, licensed technologies are also considering to operate in the unlicensed bands. The LTE-U —LTE unlicensed, a recent enhancement of LTE to boost cell coverage via the operation in the ISM band—is the best example here [6]. In the near future, communication above 60 GHz is foreseen to meet the throughput demand of the next generation wireless networks (NGWN). Hyper dense deployment of small cells will be necessary to compensate for higher path loss in these frequency ranges. In this scenario, exclusive frequency assignment per technology is beyond feasibility, thus the need for autonomous DSA mechanisms.

Detecting signals of other technologies is the prerequisite of DSA. The most straightforward approach is to sense the presence of energy in the medium, commonly referred to as energy detection [7,8,9,10]. Sometimes, merely detecting the signal’s existence is not sufficient; it is also crucial to identify the technology type. In the context of vertical spectrum sharing, if the detected signal is primary, the secondary user should back off, otherwise the delay is unnecessary. For horizontal spectrum sharing, recognizing the technology provides extra insights to counteract the interference: e.g., for a Wi-Fi network, if the concurrent technology is LTE-U [11], it would be beneficial to maximize the usage of the periodical off-time (LTE-U switches off traffic periodically so that Wi-Fi can share the medium). From a broader perspective, real-time technology recognition is essential for the interoperability among Multiple Radio Access Technologies (Multi-RAT) in the NGWN [12,13].

Mainstream technology identifiers often share the following limitations: (i) the requirement for a priori knowledge of the target technology (i.e., the known pattern inside the signal of the target technology, such as the preamble or training sequence during the data transfer), making it difficult to distinguish multiple technologies in a single implementation; (ii) the requirement for the RF signal to be sampled above Nyquist frequency; else a certain part of the signal will be “cut off”, making it impractical to be identified by techniques such as matched filtering or preamble detection; (iii) the involvement of long-term observation and intensive computation is not suitable for real-time applications on embedded devices; finally, (iv) most solutions can identify technologies that operate either purely in streaming mode (likely in the licensed spectrum) or in non-streaming mode (likely in ISM bands); hence, they are insufficient to achieve DSA across the spectrum as a whole.

To overcome these constraints, this work presents technology recognition in both licensed and the unlicensed spectrum using novel features from RSSI distribution and time domain statistics acquired at a sub-Nyquist sampling rate. As the analysis of RSSI is straightforward, technology independent and less demanding in terms of system requirements, it is more suitable for achieving technology recognition on constrained devices. Note that we consider constrained devices to be the equipment with no more than 50 kB RAM and 250 kB nonvolatile memory (e.g., flash), and has limitations on energy consumption [14].

The remainder of this paper is organized as follows: Section 2 discusses the related work; Section 3 explains how the properties of signals affect the characteristics of the RSSI distribution; next, the RSSI of representative technologies (Wi-Fi, LTE, DVB-T and Bluetooth) are explored in Section 4, followed by a thorough study of the appropriate feature space; then, the usage of novel features from the RSSI distribution is illustrated with a sample algorithm and experimentally validated in Section 5; finally, Section 6 concludes this paper and proposes directions for future work.

## 2. Related Work and Contributions

### 2.1. Technology-Specific Detection Solutions

Matched-filtering, waveform-based sensing and cycle-stationary feature detection are the most known technology-specific detections [15,16]. Matched-filtering is commonly applied at the early stage of a wireless decoder to maximize the signal-to-noise (SNR) ratio. It outperforms energy detection in terms of sensitivity, however only for a given technology. Known patterns, such as preambles, are often utilized in wireless systems for the receivers to achieve coherence with the transmitters. Detection based on these patterns is highly precise, though some level of timing and carrier synchronization has to be achieved beforehand. Cycle-stationary feature detection exploits the built-in periodicity of modulated signals, which often involves autocorrelation or spectral correlation function [17]. The main advantage is its robustness to uncertainty in noise. However, it is computationally complex and requires a significantly long observation time [15]. The work in [18] leverages on the fact that modulated signals follow a Rayleigh distribution, while noise follows a Gaussian distribution, and transmitters are identified by extracting Rayleigh and Gaussian sub-components from the raw spectra. Although the primary goal of [18] is to establish a spectrum inventory for regulators, the utilization of sophisticated machine learning techniques and off-line observation makes it unsuitable for real-time applications. Given the state-of-the-art of technology-specific recognition, there is still a need to further reduce complexity and to break the interdependency between algorithms and technologies.

### 2.2. Existing Studies of the Distribution of RSSI

In contrast to the common assumption that RSSI follows a Gaussian distribution [19,20,21,22], there are many circumstances causing RSSI to form bimodal or even multi-modal probability distributions. The works in [23,24] simply mention this phenomenon without specifying causes. The work in  [25] suggests that fading is related to the irregular RSSI distribution. More concrete causes for this phenomenon are stated in [26], including the orientation of antenna, the presence of moving objects or humans. These studies have one thing in common: the research is driven by RSSI-based localization in Wi-Fi networks. Hence, the signal strength is computed based on successfully decoded Wi-Fi packets, which are primary beacons from access points. While these observations are certainly true, they are limited to the Wi-Fi technology and focus on alleviating the instability in signal strength rather than investigating it.

The RSSI study in this paper is based on raw in-phase and quadrature (IQ) samples captured by a software-defined radio (SDR) device. It proves that the multi-modal probability distribution of RSSI is also related to the characteristics of the signal itself, causing the distribution to form stable and distinctive patterns. From this aspect RSSI can be used for technology recognition, which is one of the contributions of this paper.

### 2.3. Existing Application of RSSI in Technology Recognition

RSSI is readily available on many commercial radio chipsets; hence, several pioneering studies in technology recognition on small-scale devices make use of RSSI due to practical advantages. The work in  [27] proposes to identify technologies with different medium access control (MAC) mechanisms by observing the transmission and idle period in the time series of RSSI. Two types of classifiers are presented and experimentally validated to identify sub-categories of Wi-Fi standards. The authors of [28] use support vector machines as a machine learning algorithm to identify three generic types of MAC mechanisms, by observing the transmission interval duration and the received power for each operating MAC. Building upon the observation of the transmission interval, the authors of [29] introduce the peak-to-average power ratio and hardware-specific features. For instance, the presence of a microwave oven is identified when the RSSI of the chip CC2420 drops below the noise floor, which is likely caused by saturation in the receiver chain. The intrinsic periodicity extracted by a simplified version of the spectral correlation function proposed by [30],and the characteristics of the spectrum trace obtained by a commercial Wi-Fi card proposed by [31] are used in combination with features of RSSI series (such as idle and transmission time) to distinguish technologies in the 2.4-GHz ISM band. Although each of these works has its own strength in certain application scenarios, the common interdependency on features from the time domain of RSSI series, such as packet duration and inter-packet gap, implies that they lack the capability to distinguish streaming technologies, such as LTE and DVB-T. In addition, particular approaches such as [28] relying on complex machine learning algorithms are unsuitable to implement on constrained devices.

### 2.4. Contributions

This work is complementary to existing RSSI-based technology recognition solutions, in the sense that the targeted technologies and application scenarios are different. The goal of this work is to achieve DSA capabilities for NGWN; hence, we focus on streaming technologies beyond the well-studied coexistence problem in the ISM bands. Eventually, prior solutions can be combined with this work to extend the range of recognizable technologies and reduce complexities. To the best knowledge of the authors, this work is the first effort (i) to explain the multi-modal distribution of RSSI with the properties of the signals, other than external factors such as fading, (ii) to systematically explore the features of RSSI from real-life wireless networks and (iii) to apply novel features of RSSI distribution for technology recognition, using a computationally simple and technology agnostic classification algorithm for constrained devices.

## 3. Multi-Modal Distribution of RSSI

RSSI can be obtained in various approaches. Many wireless chipsets calculate RSSI for individual received packets to indicate the link quality between certain devices. As each vendor has its own method to compute RSSI, this makes RSSI values technology and/or device specific. In this paper, RSSI is calculated as the average of the squared magnitude of samples in logarithmic scale, as shown below:

(1) $$y\left[n\right]=10\times log_{10}(\frac{1}{N}\sum_{k=1}^{N}\left(I{\left[k\right]}^{2}+Q{\left[k\right]}^{2}\right)$$

where y[n] denotes an RSSI value, I[k] and Q[k] denote the in-phase and Quadrature components of the received sample, respectively, and *N* indicates the amount of samples used to compute one RSSI. The unit of y[n] is dB, because it is uncalibrated. As the RSSI is obtained from a continuous range of samples from the analog-to-digital converter (ADC), without any filtering in favor of a specific technology, therefore it is generic.

In practice, the probability density function (PDF) is approximated by the normalized histogram. Empirically, the bimodal or multi-modal-like irregular RSSI distribution is linked to the sudden change of signal strength. It could be caused by external factors such as shading or variation in the signal itself, which can be further divided into: (i) discontinuous transmission in the time domain; and (ii) the change of the amount of allocated carriers in the frequency domain.

### 3.1. Discontinuous Transmission

**Figure 1:**
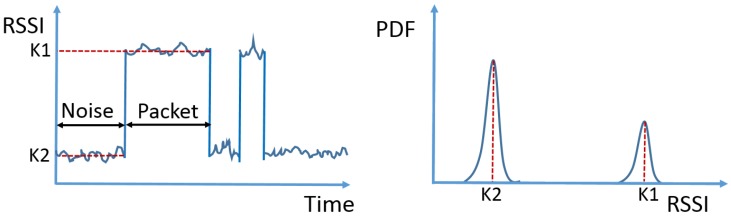
An example RSSI trace of a discontinuously-transmitted signal (**left**) and the corresponding PDF (**right**).

Discontinuous transmission is often observed from technologies in the unlicensed bands, where carrier sensing is generally required before a packet is transmitted, resulting in randomly inserted pauses between consecutive packets. An example RSSI of a discontinuous signal and the corresponding PDF are shown in Figure 1. The received signal strength reaches a stronger level (denoted as K1) when packets are being transmitted and falls back to a weak level (denoted as K2) when only noise is present. Consequently, the PDF contains two peaks: the peak on the left side is caused by noise, while the peak on the right results from the signal. Note that the values K2 and K1 are not derived by calculation. In this illustration, K2 is bigger than K1 because the medium is idle for more than 50% of the time. Additional peaks may appear in the PDF when packets are transmitted by different devices or with different power. Generally speaking, the PDF of discontinuous transmission contains multiple peaks that are situated relatively apart, and the peak at the leftmost side coincides with the distribution of the noise.

### 3.2. Variation in Carrier Allocation

**Figure 2:**
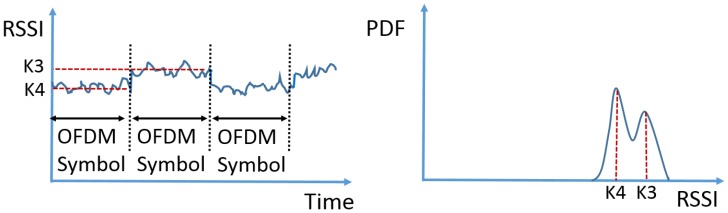
An example RSSI trace of the signal modulated with a variable amount of subcarriers (**left**) and the corresponding PDF (**right**).

The second cause of the multi-modal RSSI distribution is the variation of the amount of subcarriers. Driven by the high throughput requirement, multi-carrier modulation has gradually become the dominant modulation scheme in modern communication systems. Orthogonal frequency division multiplexing (OFDM) is undoubtedly the most popular multi-carrier modulation technique. Similar to single-carrier modulations, an OFDM symbol is transmitted for a certain amount of time; however, it has an extra degree of freedom: each OFDM symbol may occupy different amounts of subcarriers. The amount of occupied subcarriers in an OFDM symbol corresponds to the number of null subcarriers at the input of the inverse fast Fourier transform (iFFT), after which a cyclic prefix is added to form the complete OFDM symbol. The change in the quantity of occupied subcarriers causes the signal to be stronger in one OFDM symbol time than the other. An illustration of the OFDM signal in time domain and the corresponding PDF are shown in Figure 2. The example RSSI plot has two levels of average signal strength, namely K3 and K4; as a result, the PDF exhibits a bimodal distribution. Intuitively, the OFDM symbol containing more subcarriers would have a relatively stronger signal strength. This assumption is validated experimentally in the next section.

#### Experiment

A simple experiment is conducted to investigate how exactly the quantity of carriers is influencing the signal strength. The experiment involves two USRP B200 mini devices [32]. It is a small-scale SDR platform, used in combination with the GNU Radio software [33] installed on a host computer. GNU Radio provides an example implementation of the OFDM transceiver, which is used to generate the OFDM signals for this experiment. The two USRPs are used as the transmitter and receiver, respectively. To eliminate the possibility of fading, the receiver is connected via a coaxial cable to the transmitter with a 30-dB attenuator in between.

**Figure 3:**
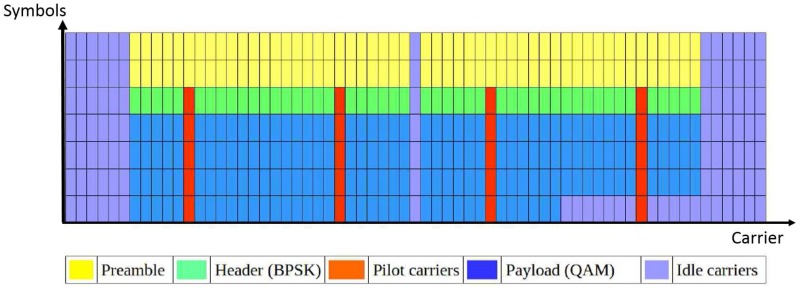
The packet structure used in the example OFDM transceiver in GNU Radio.

Figure 3 illustrates the OFDM packet structure in GNU Radio, where each row denotes an OFDM symbol in time and each column denotes a subcarrier. There are 64 subcarriers in total, among which 52 are occupied, while the rest are idle. The first two OFDM symbols are used for preamble transmission (indicated by yellow grids in Figure 3); the third symbol is used to transmit the header of the packet; while the remaining symbols contain the payload data. Except for the preamble, all of the OFDM symbols contain four pilot carriers, as indicated by the red grids. The packets are successfully decoded by the receiver, which guarantees that the signal is properly transmitted.

The simple OFDM transmitter does not have medium access control; hence, packets are transmitted continuously. Furthermore, the packet size is static, and there are no bits padded at the end of a packet. This means that the number of occupied carriers in the last OFDM symbol of a packet is dependent on the payload size. By applying the payload size of 123, 127 and 131 bytes, there are 12, 28 and 44 subcarriers occupied in the last OFDM symbol, respectively. The samples are transmitted at the rate of 200 kHz. The same rate is used to capture samples by the receiver side. An RSSI is calculated with Equation (1) for *N* = 160.

**Figure 4:**
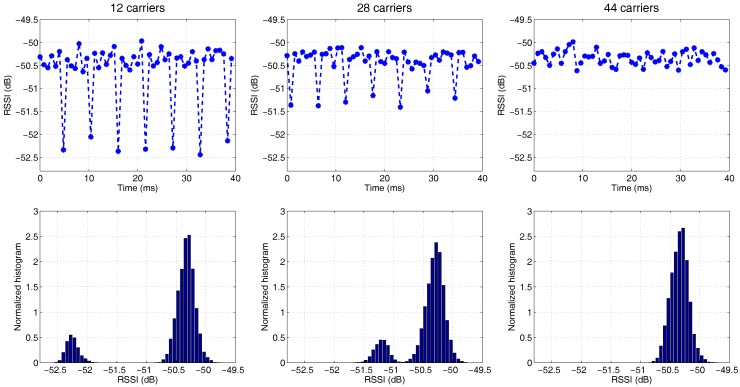
The RSSI of OFDM signals generated by GNU Radio, with a variable amount of subcarriers in the last symbol of a packet (indicated in the title of the graphs); the first row displays RSSI measurements over time, while the corresponding histograms are displayed in the second row.

The RSSI traces are shown in the first row of Figure 4. These plots illustrate that the RSSI fluctuates periodically over time, and the period corresponds to the duration of a single packet (including the header and preamble, a packet contains 14 OFDM symbols, each symbol has 80 samples, including 16 samples for cyclic prefix, hence a packet lasts for 80 × 14/200 kHz = 5.6 ms). It is observed that the signal strength of the last OFDM symbol in a packet is weaker than the rest of the packets. Additionally, the magnitude of variations becomes smaller when more subcarriers are occupied. The same conclusion can be drawn from the histogram in the second row of Figure 4. The histograms of “12 occupied carriers” and “28 occupied carriers” exhibit bimodal distributions, where the peak on the left side and that right side represent the signal strength of the last OFDM symbol and the remainder of the packet, respectively. The peak on the left side gradually moves towards the main peak with the increment of the number of occupied carriers and eventually merges with the main peak, as shown in the normalized histogram for the case of “44 occupied carriers”.

In conclusion, this simple experiment proves that for multi-carrier modulated signals, the more carriers are occupied, the stronger the signal strength gets. However, unlike the situation in non-continuous transmission, the peaks are situated relatively close to each other (only a few dB difference in between). This is because the impact on signal strength of the amount of subcarriers is significantly smaller than the impact of burst transmission. A multi-modal RSSI distribution may be formed when more flexible subcarrier allocation schemes are applied, such as the usage of resource block in LTE, which will be explained in the following sections.

### 3.3. Summary

**Figure 5:**
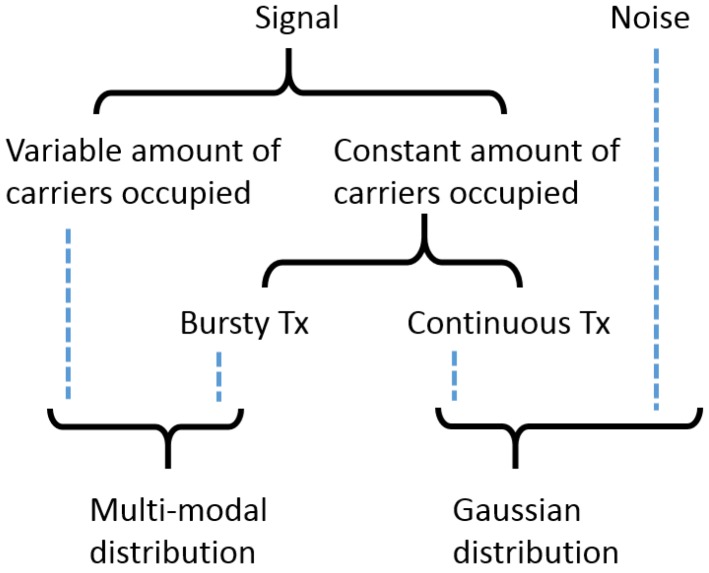
The categories of signals and the corresponding PDF.

The situations where RSSI obeys a Gaussian distribution or irregular multi-modal distributions are summarized in Figure 5. On the top level, a distinction is made between signals and noise, where signals refer to human-generated electromagnetic waves and noise refers to any uncontrollable disturbance in the RF system. Next, signals are divided based on whether the number of modulated carriers is constant or not. Note that the single-carrier modulated signal is considered as a special case of signals with a constant amount of carriers. Then, the signals modulated with constant amount of carriers are further divided based on whether the transmission is continuous. The conclusion is that RSSI forms a Gaussian distribution when the signal is not present or it is modulated with a constant amount of carriers and continuously transmitted; in all other cases, a bimodal or multi-modal distribution is observed instead.

## 4. Characterization of Real-Life Signals

A set of experiments is performed to explore how RSSI can be used to characterize signals from real-life technologies. First, the selected technology types and the motivations are introduced in Section 4.1. Then, the RSSI measurements obtained by both a high-end spectrum analyzer and a small-scale SDR device are observed in Section 4.2.1 and Section 4.2.2, respectively. Finally, the appropriate features are extracted in Section 4.3 for technology recognition.

### 4.1. Signal Selection

Real-life wireless technologies can be divided into two categories based on continuity in the time domain, namely the streaming and non-streaming technologies. Streaming technologies are mostly used in the licensed spectrum, representing technologies including DVB-T and the downlink signal of the Long-Term Evolution (LTE) technology used by the 4G cellular network; while non-streaming technologies are more often seen in the unlicensed spectrum, such as Wi-Fi, ZigBee and Bluetooth. For each of these technologies, its medium access control and carrier allocation method are briefly introduced.

In terms of the medium access scheme, Wi-Fi and ZigBee belong to the same category, because they both use carrier-sense multiple access (CSMA) to avoid collision [34], leading to random bursty signals in the time domain. Instead of CSMA, Bluetooth uses the frequency hopping spread spectrum, and different Bluetooth piconets rely on different frequency hopping sequences to share the medium, causing random bursty transmission in both the time and frequency domain. Streaming technologies such as DVB-T and LTE do not pause for medium access control.

Carrier allocation of single-carrier-based technologies is by definition constant. Hence, this paragraph only discusses OFDM-based technologies, i.e., IEEE 802.11a/g-compliant Wi-Fi, DVB-T and LTE (ZigBee uses Offset QPSK and Bluetooth uses GFSK, both being single-carrier modulation). Wi-Fi performs padding at the end of a packet to ensure that the payload size is multiples of the amount of data carriers. Similar to Wi-Fi, all carriers of OFDM symbols in DVB-T are constantly occupied; hence, there is no variation in carrier allocation for Wi-Fi and DVB-T. The LTE standard fragments the frequency-time plain into very fine resource blocks and resource elements (each resource block is 180 kHz wide and 0.5 ms long, consisting of 12 subcarriers and seven OFDM symbols, where a single carrier in an OFDM symbol is referred to as a resource element) [35]. Some resource blocks are periodically used for control and broadcasting purposes, whereas others are available to carry user data. There are 16 resource elements in a resource block used to transmit cell-specific reference signals, which are always present no matter if the resource block is allocated for data transmission or not. Thus, the LTE signal is modulated with a variable amount of carriers and transmitted in very fine and regular intervals.

Based on the above description, we select Wi-Fi, LTE and DVB-T as the target technologies to be classified in this work. The motivations are as follows: (i) the three technologies are either different in carrier allocation or medium access control, which are suitable to be identified with RSSI distribution; (ii) these technologies are relatively wideband, which are more challenging to recognize for narrow-band devices, hence more meaningful to investigate. For differentiating multiple non-streaming technologies, such as Bluetooth, Wi-Fi, and ZigBee, information in the time domain should be involved, which is briefly illustrated by a case study in Section 4.3.2. Note that identification of technologies in ISM bands with time domain RSSI-based statistics are already proposed in [27,28,29]; therefore, it is not the focus of this work. The characteristics of the three selected signals are summarized below:

- Wi-Fi (IEEE 802.11a): signal transmitted in random bursts, modulated with a constant amount of carriers;
- LTE: signal transmitted in very fine and regular intervals, modulated with a variable amount of carriers;
- DVB-T: signal transmitted continuously and modulated with a constant amount of carriers.

### 4.2. Experiments

In subsequent experiments, the Wi-Fi signal is captured in an office environment, including two access points (AP’s) at 5540 MHz (Channel 108) and on average 20 associated work stations; the LTE signal is captured from a nearby base station, operating in FDD (Frequency Division Duplex) mode at 806 MHz, around the Ghent area of Belgium; finally, the DVB-T signal is collected from the local TV broadcasting station at 482 MHz.

#### 4.2.1. Experiment with the Spectrum Analyzer

**Figure 6:**
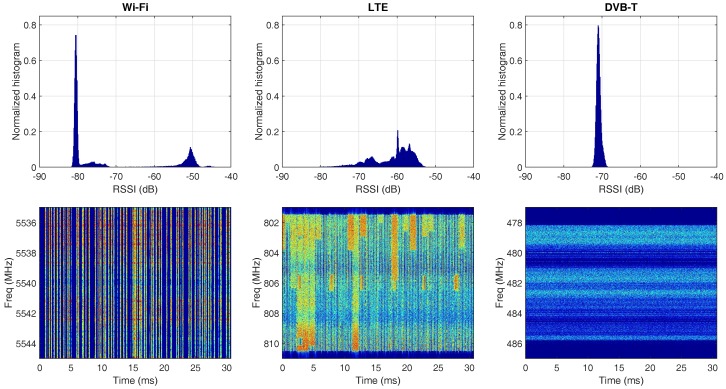
The normalized histograms of RSSI (first row) and the spectrograms (second row) for Wi-Fi, LTE and DVB-T signals. All graphs are obtained by post-processing of raw IQ samples collected by the Anritsu 2690A spectrum analyzer at the rate of 10 MHz.

An Anritsu MS 2690A spectrum analyzer [36] is used to capture samples of each of the aforementioned signal types. The samples are collected at the rate of 10 MHz for a duration of 1 s. The RSSI is calculated using Equation (1) for *N* = 200. In total, 50 k RSSI values are obtained. The first row of Figure 6 contains the normalized RSSI histograms of the three selected technologies, and the spectrograms are shown at the corresponding position of the second row. The time span of the spectrogram is limited to 30 ms for visualization purposes.

As anticipated, the Wi-Fi signal appears as short and random bursts in the spectrogram. Since the signals belong to multiple stations and AP’s, the histogram contains several peaks between −50 dB and −75 dB, corresponding to signals transmitted by nearby and remote stations, respectively. Note that the histogram varies with the traffic load of the Wi-Fi network and the distance to the stations and AP’s. However, due to the nature of contention-based collision avoidance, there is always a significant amount of noise samples present in the time frame of 1 s. Hence, the high peak on the left side of the histogram contributed by noise always exists.

The LTE signal has the most versatile spectrogram, which is primarily occupied by the frequently occurring reference signal in the idle resource blocks. The 1 MHz-wide signal occurring every 5 ms at the center of the LTE band is the synchronization sequence. The remaining parts of the spectrogram with higher intensity are the resource blocks active for data transmission. The histogram of the LTE signal contains multiple peaks spreading out from −80 dB to −55 dB. Unlike the histogram of Wi-Fi, it is rather difficult to map the peaks to the exact causes for LTE. Moreover, the LTE signal is highly sensitive to the instant throughput demands of the users; thus, the magnitude of the peaks varies from one moment to another. What remains unchanged is the extremely wide range of variation in the signal strength and the large number of peaks present in the histogram.

The DVB-T’s spectrogram shows that it is highly stable in both the time and frequency domain. This is because the current DVB-T standard only allows continuous transmission and does not contain flexibility in carrier allocation. Therefore, its histogram simply matches the characteristic of Gaussian distribution: only one narrow peak is present. Sometimes, fading may cause some tiny peaks to appear next to the main one. In general, the RSSI’s histogram from the DVB-T signal has the minimum number of peaks and the least amount of variation in signal strength among the three technologies.

#### 4.2.2. Experiment with Small-Scale RF Devices

The previous experiment gives promising results for signal recognition based on the RSSI distribution. However, the results are obtained by a spectrum analyzer, which is capable of much more power analysis, and the added value of RSSI-based signal recognition for such a high-end device is rather trivial. Additionally, doing measurements with a spectrum analyzer is not always practical and cost efficient. Therefore, the preferred implementation platform is small-scale and low-cost RF devices. However, this raises another doubt: will the RSSI measurements obtained by low-end devices have sufficient diversities for signal recognition? In fact, the 10-MHz sample rate used in the last experiment is above the limit of many inexpensive devices (e.g., ZigBee sensors or Bluetooth dongles).

**Figure 7:**
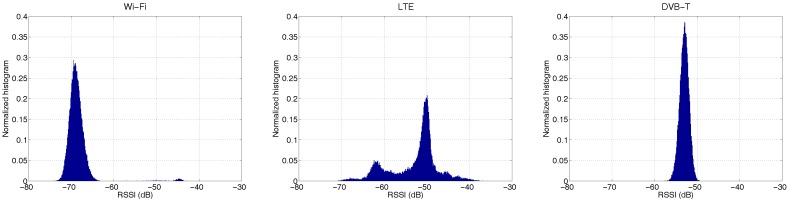
The normalized histograms of RSSI for Wi-Fi, LTE and DVB-T signals obtained by USRPB200 mini with the sample rate of 1 MHz.

To answer this question, we repeat the previous experiment with a low-end SDR device (USRP B200 mini) at the sample rate of 1 MHz. The amount of samples used to compute a single RSSI (denoted by *N* in Equation (1)) is scaled down to 20. In total, 50 k RSSI measurements are collected during the time frame of 1 s. Since a spectrogram of 1 MHz wide contains limited information, only the histograms of the RSSI measurements are displayed in Figure 7.

At first sight, the RSSI measurements from the USRP are stronger than the ones from the spectrum analyzer. Taking the most static signal DVB-T as an example, the average RSSI measured by USRP is around −54 dB, while only −72 dB is obtained by the spectrum analyzer. Since the RSSI measurements are not calibrated, many factors (e.g., hardware, gain) can contribute to the difference, which are out of the scope of this paper. The important message here is that the histograms obtained by USRP at a lower sample rate are highly consistent with their counterparts obtained by the spectrum analyzer. Therefore, RSSI measurements obtained at a much lower sampling rate, by small-scale devices, also have potential to be applied for signal recognition.

### 4.3. Feature Space Design

Simple and reliable features are essential for automatic technology recognition on constrained devices. We limit the selection of features to narrowband RSSI. This means features from the wideband spectrogram are excluded, due to its requirement for both the RF front end capability and the baseband processing power. In the subsequent sections, we first discuss features extracted from the RSSI distribution (i.e., histogram) to distinguish Wi-Fi, DVB-T and LTE; then, supplementary features obtained from time domain RSSI series are introduced for categorization among multiple non-streaming technologies.

#### 4.3.1. Features from the RSSI Distribution

**Figure 8:**
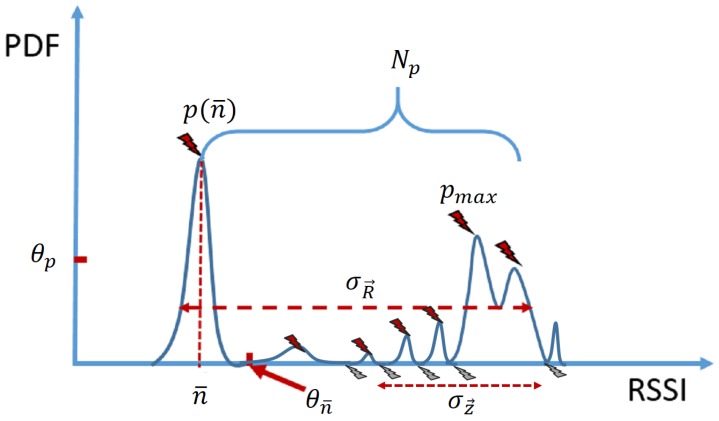
An illustration of the features extracted from the distribution of RSSI.

Based on the previous observation of the RSSI distribution, we select several features that have potential for automated technology recognition, as illustrated in Figure 8 and explained as follows:

- $\sigma_{\vec{R}}$: the standard deviation of the RSSI vector $\vec{R}$. It indicates the range of variation in the signal strength.
- $N_{p}$: the number of peaks in the histogram of RSSI is a simple way to describe the shape of the distribution. A point in the histogram is recognized as a peak when it is above its two neighboring points.
- $\bar{n}$: the average power level of the noise, which corresponds to the location of the leftmost peak in the histogram, and it should be situated to the left of a certain threshold, denoted as $\theta_{\bar{n}}$.
- $p(\bar{n})$: the probability that the measured RSSI is equal to the average noise power level $\bar{n}$, i.e., $p\left(\bar{n}\right)=P\left(RSSI=\bar{n}\right)$. This is the amplitude of the noise peak in the RSSI histogram, which is proportional to the amount of time the signal is interrupted. It is identified when the peak corresponding to $\bar{n}$ is above $\theta_{p}$.

The relationship between the selected features $\sigma_{\vec{R}}$, $N_{p}$, $p(\bar{n})$ and the technologies is summarized in Table 1. The feature space of the RSSI histogram associates each of the selected technologies with at least one stable feature, making the automated signal recognition rather straightforward. The rest of the features (denoted as $\sigma_{\vec{z}}$ and $p_{max}$ in Figure 8) are elaborated in Section 5.3 when mixed signals are also considered as a signal class.

#### 4.3.2. Features from RSSI Time Series

As seen in Table 1, Wi-Fi’s distinctive feature is the visible noise peak $p(\bar{n})$, which is a common feature of non-streaming technologies. Therefore, how can we distinguish multiple non-streaming technologies with narrowband RSSI? We aim to answer this question with a case study of Bluetooth and Wi-Fi.

**Figure 9:**
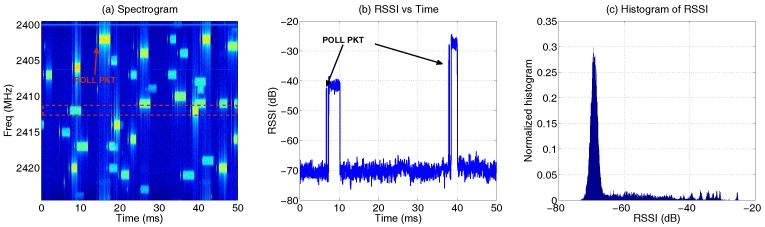
Characteristics of the Bluetooth signal: (**a**) the 25 MHz-wide spectrogram of the Bluetooth signal; the 1-MHz signals on the middle channel are extracted to obtain the RSSI, as shown in (**b**); (**c**) the normalized histogram of the narrowband RSSI.

First, IQ samples are collected at 25 MHz for 1 s when 10 pairs of Bluetooth piconets are activated in a testbed environment. Note that piconet refers to a group of connected Bluetooth devices, which has a specific frequency hopping sequence determined by the ‘master’ device. More experiment details are described in [37]. Figure 9a displays 50 ms of the recorded signal in the form of a spectrogram. As expected, the Bluetooth signal is spread randomly over multiple channels, and there is also variation in packet duration and signal strength. The latter is due to the fact that the Bluetooth piconets are placed at different distances from the sample collector. The variation in packet duration is according to the Bluetooth standard [38]. Bluetooth piconet may hop to a new channel every 625 μs, which is the base of the transmission slot. A Bluetooth data packet may last 1, 3 or 5 slots, depending on the payload length (note that *N* × 625 μs is the maximum duration of an N-slot packet, not the exact time). In addition, various control packets are sent for link management purposes, among which a short probing packet (approximately 126 μs long, referred to as the POLL packet) is always sent from the master to the slave to initiate data transfer, as indicated in the Figure 9.

Next, the 25 MHz-wide IQ samples are down sampled to 1 MHz to derive RSSI with Equation (1) when *N* = 20. The down sampling is combined with proper filtering, so that only signals in the middle channel (indicated by the red rectangle in Figure 9a) remain. The RSSI vector of the first 50 ms is plotted in Figure 9b, and the normalized histogram of the RSSI vector is shown in Figure 9c. As expected, the histogram of 1-MHz RSSI from the Bluetooth signal also contains a high $p(\bar{n})$ like Wi-Fi. The histogram appears to have a more uniform distribution in the interval of [−60, −20] dB than Wi-Fi, because in this particular setup, the Bluetooth network has more transmitters spread over a larger area than Wi-Fi. Clearly, the observation is subject to the measurement setup; hence, it should not be generalized to distinguish Wi-Fi and Bluetooth.

**Figure 10:**
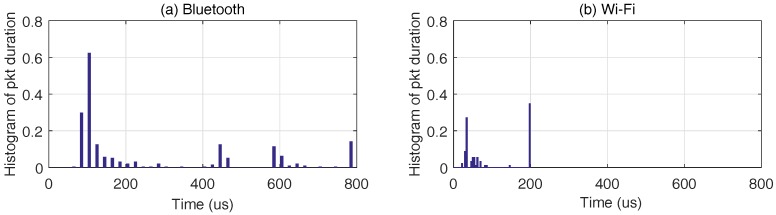
The normalized histograms of packet duration of Bluetooth and Wi-Fi, derived by applying threshold of −65 dB to the RSSI traces, which are obtained at a 1-MHz sampling rate and averaged over 20 μs. The scale of the graph is zoomed to the range [0, 800] μs for visualization purposes.

The histogram of Bluetooth RSSI confirms that simply using the features of the RSSI distribution is insufficient to categorize non-streaming technologies, thus the need for other features. Intuitively, by setting an energy threshold λ and counting the number of consecutive RSSI above the threshold, one can obtain the duration of a packet in the air as follows:

$$T_{P_{i}}=N_{i}\times T_{RSSI},N_{i}\in Z.$$

where $N_{i}$ is the number of RSSI corresponding to the *i*-th packet and $T_{RSSI}$ denotes the averaging interval of a single RSSI, in this case $T_{RSSI}=20\;\mu$s. $N_{i}$ is identified based on the following condition $y_{i,j}>\lambda$ and $j=1,2,\ldots ,N_{i}$, where $y_{i,j}$ denotes an RSSI with index *j* in the frame of the *i*-th packet. When this process is iterated through the entire RSSI trace, we obtain a vector $\vec{T_{P}}$ containing the duration of all packets in the trace. The condition λ=−65 dB is applied to the RSSI traces to derive $\vec{T_{P}}$, and the normalized histograms of packet duration (denoted as $H(\vec{T_{P}})$) of the Bluetooth and Wi-Fi are displayed side by side in Figure 10. The Bluetooth’s $H(\vec{T_{P}})$ shows a peak at about 120 μs, and this corresponds to the duration of the POLL packet; while Wi-Fi’s $H(\vec{T_{P}})$ shows a peak around 20 to 40 μs, which corresponds to the duration of an acknowledgment packet [27]. Hence, the two technologies can be told apart by observing the peaks’ location in $H(\vec{T_{P}})$.

As demonstrated in this case study, the time domain features such as packet duration $T_{p}$ can be readily extracted from narrowband RSSI; Furthermore, these features are determined by the specific wireless standard, which are immune to the change of measurement scenarios, hence being reliable for classification of non-streaming technologies.

## 5. Automatic Signal Recognition

Given that prior works [27,29,30,31] were dedicated to recognizing ISM band (non-streaming) technologies using time domain features, the remainder of this work aims to automate technology recognition using a different feature space established from the RSSI distribution, to identify a different set of technologies, including both non-streaming technologies (represented by Wi-Fi) and streaming technologies beyond the ISM bands (i.e., DVB-T, LTE). The usage of the feature space is illustrated via a sample algorithm in Section 5.1 and evaluated in Section 5.2; then, the algorithm and feature space are extended for recognition of the mixed LTE-U and Wi-Fi signal in Section 5.3.

### 5.1. Sample Algorithm

The sample algorithm is summarized in pseudo-code as shown in Algorithm 1. First, Wi-Fi is recognized if the RSSI contains a large amount of noise and has s sufficient standard deviation; the latter condition is used to distinguish Wi-Fi signal from noise. Next, the LTE signal is identified when the standard deviation is above a certain threshold. Then, when the previous condition is not met and the number of peaks in the RSSI histogram is below a certain threshold, the signal is considered to be DVB-T; otherwise, it is classified as unknown. The sample algorithm relies on several thresholds, which are derived via an independent training dataset in the following way. Note that this is essentially a process to “learn” the optimal threshold with a supervised algorithm.

- $\theta_{\bar{n}}$ is the upper bound of the average noise level, obtained by the maximum of $\bar{n}$ in the collected Wi-Fi traces plus the standard deviation of $\bar{n}$.
- $\theta_{p}$ determines the minimal amount of noise present in the Wi-Fi’s RSSI measurements. It is calculated by the smallest ‘noise peak’ minus the standard deviation of the noise peaks in the Wi-Fi’s RSSI measurements.
- min(Wi-$Fi.\sigma_{\vec{R}})$ denotes the minimum standard deviation among the collected RSSI measurements of Wi-Fi, which is used to differentiate Wi-Fi from noise.
- $(min\left(LTE.\sigma_{\vec{R}}\right)+max(DVB$-$T.\sigma_{\vec{R}}))/2$ denotes the medium of the minimum and the maximum standard deviation of LTE and DVB-T’s RSSI measurements. It is used to differentiate LTE and DVB-T signal.
- max(DVB-$T.N_{p})$ denotes the maximum number of peaks in the histograms of the RSSI measurements of DVB-T. It is used to exclude unknown signals from the DVB-T signals.

**Algorithm 1** RSSI distribution-based technology recognition.**Intput:** $\vec{R}$ // a vector of RSSI measurements**Output:** sig // the identified signal type**Function:** // derive variables from $\vec{R}$ $\vec{H}\leftarrow Histogram\left(\vec{R}\right)$ $\sigma_{\vec{R}}\leftarrow StdDeviation\left(\vec{R}\right)$ $\bar{n}\leftarrow LocOfLeftmostPeak\left(\vec{H}\right)$ $p\left(\bar{n}\right)\leftarrow AmpOfLeftmostPeak\left(\vec{H}\right)$ $N_{p}\leftarrow TotalNumOfPeaks\left(\vec{H}\right)$  // process to make decision **if** $\bar{n}\leq \theta_{\bar{n}}$ & $p\left(\bar{n}\right)\geq \theta_{p}$ **then**  /**********************************************/  **if** $\sigma_{\vec{R}}\geq min(Wi$-$Fi.\sigma_{\vec{R}})$ **then**   sig← Wi-Fi  **else**   sig← noise  **end if**  /**********************************************/ **else**  **if** $\sigma_{\vec{R}}\geq (min\left(LTE.\sigma_{\vec{R}}\right)+max(DVB$-$T.\sigma_{\vec{R}}))/2$ **then**   sig← LTE  **else**   **if** $N_{p}\leq max(DVB$-$T.N_{p})$ **then**    sig← DVB-T   **else**    sig← unknown   **end if**  **end if** **end if**


### 5.2. Validation

Additional measurements are collected at three locations, in the early afternoon of two consecutive days. The target signals are identical as before, namely the Wi-Fi office network at 5540 MHz, the LTE downlink signal at 806 MHz and the DVB-T signal at 482 MHz. All measurements are conducted in an office building of 12 × 80 m. To increase the diversity of signal strength, the measurement locations are placed on the north, east and west side of the building, respectively. Each day, 10 traces are collected per location and per technology. A trace contains $1\times 10^{6}$ IQ samples, obtained by USRP for a duration of 1 s, at the ADC sample rate of 1 MHz. Note that 1 MHz falls within the bandwidth capability of common small-scale wireless devices, such as ZigBee (2 MHz) and Bluetooth (1 MHz). In total, 180 traces are collected, consisting of 60 traces per technology.

RSSI is derived according to Equation (1). First, the performance for N=20, during the observation time of one second, is analyzed. Then, we extend the evaluation for N∈[20,320], incremented at the step of 20. In our solution, large *N* corresponds to a longer average interval, hence lower update frequency of the RSSI measurements. The analysis for variable *N* is important, as developers on constrained devices usually do not have direct access to the raw IQ samples, so RSSI is provided by accessing a register or other types of interface towards hardware modules, which have a limited access rate. For instance, the TelosB sensor platform allows the RSSI register to be read at the rate of 32.15 kHz after customization [29], while the solution in [30] only refreshes RSSI at 11.3 kHz. Note that this is the rate at which the RSSI register can be accessed, it is not the sample rate used by the ADC on the RF front-end. Therefore, how often RSSI needs to be updated has a strong impact on the feasibility of implementing the solution on constrained devices. Additionally, we also evaluate the impact of the total observation time—the time interval during which the RSSI series are derived, denoted as *T*—on the detection accuracy. This is helpful to explore the minimum waiting time needed for the algorithm to achieve a given recognition accuracy.

We use three-fold cross-validation to evaluate the performance for all of the chosen *N* and *T* settings. In each round of the validation, the RSSI traces of a given technology are divided into two parts: 70% of the data are used as training data to derive thresholds used in the algorithm, while the remaining 30% are used to validate if the signal is correctly classified. This process is repeated three times; each time, different portions of the traces are used for training and validation, respectively. For each technology, the collected traces are numbered chronologically from 1 to 60, and the ranges of testing data on the three validation rounds are [1, 18], [19, 36], [37, 54].

#### 5.2.1. Analysis of *N* = 20, *T* = 1 s

The recognition results are averaged over three rounds and presented in the form of a confusion matrix in Table 2, where a row represents the actual technology type of a given RSSI trace and a column represents a technology type determined by the algorithm. For all three technologies, the probability of a true positive in the confusion matrix is above 90%, which proves that the predicted technology types are highly consistent with the actual ones. All RSSI traces of LTE signals are correctly classified, whereas 1.85% of the DVB-T and 1.85% of WiFi traces are falsely predicted as LTE, leading to in total 3.7% false positives for LTE. This indicates that the thresholds for determining $\sigma_{\vec{R}}$ and $p(\vec{\bar{n}})$ can be improved to more accurately distinguish LTE from the other technologies.

In Table 2, the most prediction errors appear for the case of Wi-Fi. This is because Wi-Fi is an indoor technology, consisting of multiple transmitters spread over the measurement sites, making its RSSI most sensitive to the influence of locations. As a matter of fact, one measurement location is relatively far from the APs and has no work station in the immediate surroundings. Consequently, the RSSI traces obtained at this location have smaller standard deviations. When none of the traces from this location are included in the training data, the probability to classify them as noise increases, which explains the high false positive rate of noise. Likewise, when the training data primarily consist of RSSI obtained at a remote location, the percentage of noise samples in the training data rises, leading to a higher $\theta_{p}$. The increased threshold is likely to exclude the traces obtained at busier Wi-Fi areas, causing them to be further classified as either LTE or unknown signal.

#### 5.2.2. Analysis of Variable *N*

**Figure 11:**
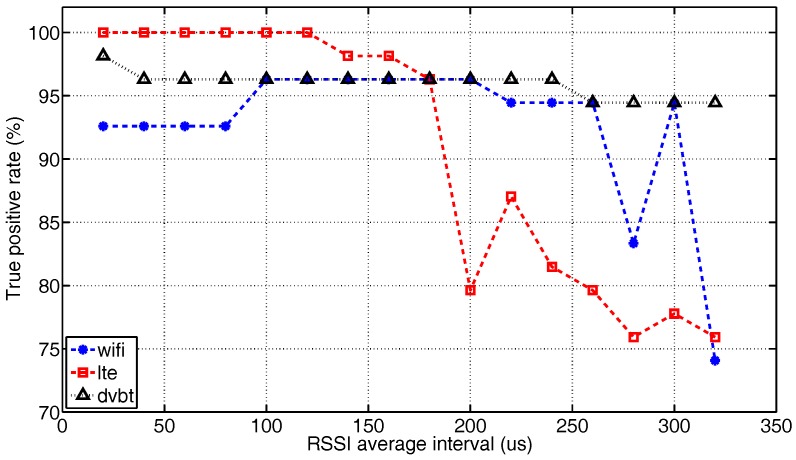
The true positive rate versus the average interval of RSSI measurements for *T* = 1 s. Because the IQ samples are acquired at 1 MHz, the interval $\frac{N}{1\;\mathrm{MHz}}$ has the same numeric value as the window size *N* in μs.

The previous analysis details the performance of the algorithm in the form of a confusion matrix. Ideally, confusion matrices for each case of *N* (i.e., the number of IQ samples used to derive an RSSI) should be presented for a full-scale analysis. However, that would introduce too many details and make it hard to perform an effective comparison. Hence, only the true positive rate of each technology is plotted in Figure 11. All results are obtained when *T* = 1 s. Given the 1-MHz ADC sample rate, the average interval $\frac{N}{1\;\mathrm{MHz}}$ has the same numerical value as *N* in μs.

Despite some local fluctuations, there is a clear trend of performance degradation due the increment of *N*. This is because the larger average interval filters out the fast changes of RSSI in the time domain. As a result, the standard deviation is reduced, which is the main feature used to identify LTE and Wi-Fi. When the averaging interval reaches 160 μs (approximately two-times the LTE symbol time) the recognition accuracy for LTE starts to drop drastically, due to the incapability to distinguish signal variations between symbols. The detection rate for Wi-Fi remains above 90% until the interval reaches 280 μs, which is approaching the duration of a Wi-Fi beacon packet. The large average window blurs the boundary between noise and signal, which makes the noise peak in the histogram less pronounced, and consequently, the recognition of Wi-Fi becomes more difficult. As DVB-T’s signal is stable in the time domain, its recognition performance is least affected by the averaging length. However, for a range of IQ samples collected in a given time frame, the larger average windows reduce the quantity of RSSI. From the statistical point of view, training processes based on a smaller population are less reliable. Hence, the recognition rate of DVB-T also decreases eventually.

#### 5.2.3. Analysis of Variable *T*

**Figure 12:**
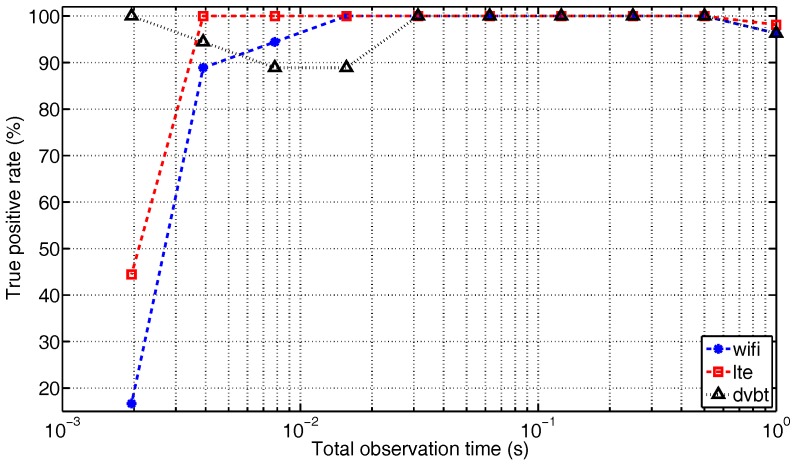
The true positive rate versus the total observation time, for *N* = 160.

Figure 12 depicts the true positive rate of each technology with respect to the total observation time *T* for *N* = 160. *T* is varied exponentially, according to $T={\left(\frac{1}{2}\right)}^{n}$ second, where n=0,1,…,9. Generally speaking, the recognition accuracy decreases when the observation time is shortened. When a comparison is made among technologies, the recognition rate of Wi-Fi is most sensitive to the variation of *T*. This is logical, as the Wi-Fi signal consists of random bursts, and during a shorter interval, the chances to capture Wi-Fi packets decrease, hence the higher probability of being categorized as noise. The shrinking of observation time does not seem to hinder LTE’s recognition until it hits 2 ms, which is equal to the duration of the two LTE subframes and represented by merely 12 RSSI data points—for *N* = 160, round (2 ms/160 μs) = 12 . In this case, the RSSI sequence does not have sufficient diversity to make a distinction between LTE and DVB-T any more. DVB-T’s recognition is rather independent of the observation time, again thanks to its stable feature in the time domain. However, there are some local fluctuations, due to the fact that it is more difficult to make a reliable prediction based on a smaller amount of RSSI data points in a short duration.

#### 5.2.4. Analysis of Practicality

In general, the experimental validation gives promising results for identifying wideband technologies on narrow-band devices. The detailed study of the confusion matrix exposes some limitations of the sample algorithm, such as the difficulty to cope with the variations of the Wi-Fi signal in the indoor environment. These limitations indicate the importance of sufficient diversities in the training data and eventually the need for more dynamic algorithms. The analysis of the true positive rate with respect to *N* and *T* unveils the conditions for the features to stay reliable, which is when the average interval of RSSI is kept below 200 μs and the observation time is above 50 ms. Recall that the sensor platforms used by [29,30] allow RSSI to be collected at 11.3 kHz and 31.25 kHz, respectively. The 200 μs ($\frac{1}{200\;\mu s}=5$ kHz) requirement can clearly be supported on these devices. The work in [29] states a minimum observation time of 2.9 ms, which suffices for the purpose of identifying only the ZigBee signal (the minimum packet interval of ZigBee is 2.8 ms). Solutions aiming for classification of multiple technologies generally rely on longer observation periods, ranging from 100 ms [31] to several seconds [30], or multiple days and months if the solution is intended for non-real-time applications [18]. Therefore, the period of 50 ms is considerably shorter than most existing solutions and hence suitable for making real-time decisions in the context of dynamic spectrum sharing.

Note that we deliberately choose not to involve direct comparison of recognition accuracy, because there is no fair way to compare solutions aiming to distinguish different sets of technologies. Instead, we focus on the system requirements (e.g., ADC sample rate, RSSI update interval), to demonstrate the feasibility of integrating the features of RSSI distribution with existing solutions.

In addition to system requirements, the complexity and scalability are also important aspects for constrained devices. To ease the complexity analysis, we decouple the sample algorithm into three stages: (i) derive variables directly from the input $\vec{RSSI}$ such as $\vec{H}$, $\sigma_{\vec{R}}$; (ii) derive variables from the RSSI histogram $\vec{H}$ such as $\bar{n}$, $p(\bar{n})$, $N_{p}$; (iii) make a decision based on the previously-derived variables. For the first stage, the histogram and standard deviation of RSSI can be obtained by simple loops with complexity $O(sizeof\left(\vec{RSSI}\right))$, where the upper bound of the execution time is linear to the input vector size. For the second stage, the iterations required to search and count peaks in $\vec{H}$ depend on the number of bins in the histogram, which is an internal setting within the algorithm; hence, the complexity is denoted as O(1), meaning the worst case execution time of the second stage is independent of the input size. For the third stage, the decision making is purely rule-based conditional statements, and the complexity has clearly no dependency on the input size, thus again O(1). Regarding scalability, the sample algorithm is not varied upon a change in the network environment. For a larger input vector size, the accuracy of the algorithm is higher, but at the cost of an increased observation period as stated in Section 5.2.3. Hence, the sample algorithm has rather low complexity and high scalability.

### 5.3. Extended Validation for Mixed Signals

It is validated that the features of the RSSI distribution can be used to categorize Wi-Fi, LTE and DVB-T when the signals are captured individually. This section extends the validation by taking into account mixed signals from multiple technologies. The chosen scenario is the operation of LTE in ISM bands. Section 5.3.1 discusses how the dataset is obtained for the mixed LTE-U and Wi-Fi signals; then, Section 5.3.2 shows how the feature space and algorithm are extended accordingly to cope with the mixed signal as a new signal class; finally the analysis of additional results is presented in Section 5.3.3.

#### 5.3.1. Dataset Extension for Mixed LTE-U and Wi-Fi Signals

As LTE-U is not yet commercialized, capturing it directly from the operator is not an option. Hence, traces containing mixed RSSI samples are fabricated based on the previously collected data. Following the recent standardization of LTE License Assisted Access (LAA) [39], the eNodeB applies carrier sensing and random back off within a certain contention window, and the transmission burst may take place for 2 ms, 3 ms, 8 ms or 10 ms, depending on the channel access priority. This ensures that Wi-Fi or other technologies could also compete for the medium. In principle, there will be no over-the-air collision; hence, the mixed trace is simply composed of interleaving Wi-Fi and LTE down link signals, collected at the same location and on the same day. In total, 60 mixed traces are produced, 10 traces for each combination of the collection date and location.

**Figure 13:**
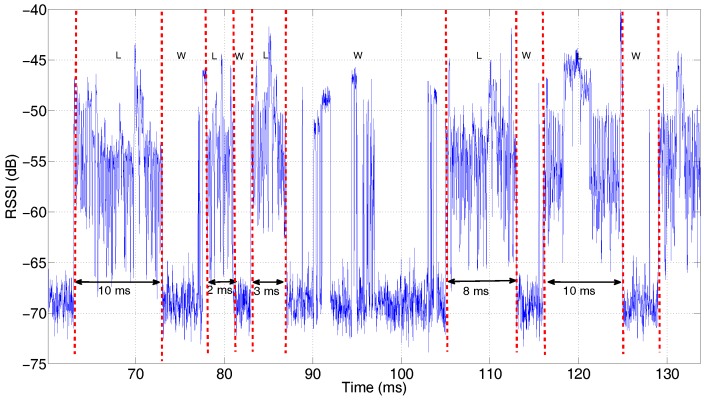
An illustration of the fabricated RSSI trace, consisting of arbitrarily interleaved RSSI from Wi-Fi (indicated by ‘W’) and LTE (indicated by ‘L’). Note that the duration of continuous LTE RSSI is chosen within (2, 3, 8, 10) ms to be compliant with the recent LTE License Assisted Access (LAA) specification.

A part of the mixed RSSI trace is shown in Figure 13 for illustration purposes. A transmission burst of LTE (indicated by ‘L’) is a continuous range of RSSI randomly selected (random permutation is used to ensure that no sample is reused) from the LTE down link samples, whereas the length of the transmission burst is chosen among the specified values (2, 3, 8, 10) ms with equal probability. Similarly, RSSI from Wi-Fi traces (indicated by ‘W’) are also selected and inserted arbitrarily into the trace, though without the limitation on the transmission duration. The fabricated traces are only based on *N* = 20 and *T* = 1, the smallest averaging interval and largest observation time, to ensure that the duration of the transmission burst from LTE is accurate.

#### 5.3.2. Algorithm and Feature Space Extension

Ideally, one should be capable of making a distinction between Wi-Fi and LTE-U within the mixed traces. However, this is only meaningful when the observation period is much shorter than the on period of LTE-U, otherwise the RSSI collected in one observation duration will easily span several appearances of LTE-U and Wi-Fi, making the classification meaningless. Currently, observation periods are in the range of 1 ms to 1 s (see Section 5.2.3), which are comparable or much longer than the LTE-U on period. Though it is possible to further reduce the observation time, this action would sacrifice the recognition accuracy by reducing in RSSI sample size. In addition, we believe it is more beneficial for the constrained devices to know the present technology types at a larger time scale, rather than the sub-millisecond precision, since the devices have limited reaction speed. Hence, instead of considering adding LTE-U as an independent signal class, the mixed signal is considered as a new class ‘MIX_LTE_Wi-Fi’. In this way, we gain more insights into the interference type while avoiding requirements in short observation and reaction time, making it more practical to apply on constrained devices.

The operation of LTE-U is adapted to non-streaming mode for fair spectrum access. This means that the mixed signal will have a noise peak just like Wi-Fi; thus, the basic algorithm can already tell it apart from LTE and DVB-T. The only addition needed is the ability to distinguish the mixed signal from the pure Wi-Fi signal. To this end, two additional features are included, as shown in Figure 8 and displayed below:

- $\sigma_{\vec{z}}$: the standard deviation of the locations (indicated by grey flashes in Figure 8 and represented by vector $\vec{z}$), where the histogram elements located to the right side of $\theta_{\bar{n}}$ are equal to zero. It indicates the amount of discontinuities in the RSSI histogram.
- $p_{max}$: the amplitude of the highest peak in the histogram apart from the noise peak.

According to [39], LTE-U only considers downlink; hence, only one sender is present, causing the RSSI distribution to have many densely-located peaks. While Wi-Fi almost always has multiple distributed senders, the RSSI distribution often contains widespread and independent peaks. This difference is well represented by $\sigma_{\vec{z}}$. Furthermore, the mixed signals tend to occupy the medium more than Wi-Fi alone, leading to smaller $p(\bar{n})$ and bigger $p_{max}$. A simple voting algorithm is formed based on these conditions to distinguish Wi-Fi and MIX_LTE_Wi-Fi, as shown in Algorithm 2. This code replaces the part in between the ‘star’ separation lines in Algorithm 1.

**Algorithm 2** Extension to distinguish Wi-Fi from MIX_LTE_Wi-Fi.**Variables:** vote← //A variable to count the vote for the mixed signals**Function:** vote=0; **if** $\sigma_{\vec{z}}\leq max(MIX\_LTE\_Wi$-$Fi.\sigma_{\vec{z}})$ **then**  vote←vote+1 **else**  vote←vote−1 **end if** **if** $p_{max}\geq min(MIX\_LTE\_Wi$-$Fi.p_{max})$ **then**  vote←vote+1 **else**  vote←vote−1 **end if** **if** $p\left(\vec{n}\right)\leq max(MIX\_LTE\_Wi$-$Fi.p(\vec{n}))$ **then**  vote←vote+1 **else**  vote←vote−1 **end if** **if** vote>0 **then**  sig←MIX_LTE_Wi-Fi **else**  **if** $\sigma_{\vec{R}}\geq min(Wi$-$Fi.p_{max})$ **then**   sig← Wi-Fi  **else**   sig← noise  **end if** **end if**


The new algorithm requires the supervised learning process of certain thresholds to be readjusted. Originally, $\theta_{\bar{n}}$ was extracted from the Wi-Fi trace, since only Wi-Fi has RSSI at the noise level. Now that the new signal class also contains the noise peak, the process to determine thresholds must adapt to recognize noise inside the mixed traces. Hence, $\theta_{\bar{n}}=max\{$Wi-Fi$.\theta_{\bar{n}}$, MIX_LTE_Wi-Fi$.\theta_{\bar{n}}\}$, and in the same way, $\theta_{p}$ is modified to be min{Wi-Fi$.\theta_{p}$, MIX_LTE_Wi-Fi$.\theta_{p}\}$.

#### 5.3.3. Result Analysis

The mixed RSSI traces are combined with the existing ones to verify the extended features and algorithm. A similar three-fold cross-validation process is applied, and the new confusion matrix is displayed in Table 3. As expected, the true positive rates of LTE and DVB-T are identical as shown in Table 2, since neither the threshold learning procedure, nor the algorithm changed for these signal classes. The true positive rate of the mixed signal is 98.15%, which shows that the extended algorithm and feature space are well capable of identifying the mixed signals from the rest of the signal classes. Interestingly, the true positive rates of Wi-Fi obtained by the new algorithm are actually higher than before. This can be better understood when looking at the false positives: Wi-Fi signals are no longer recognized as LTE or noise, thanks to the relaxed thresholds $\theta_{\bar{n}}$ and $\theta_{p}$; instead, all of the Wi-Fi traces that failed to be recognized are now identified as the mixed signal, and 1.85% of mixed traces are falsely determined to be LTE.

## 6. Conclusions and Future Work

Received signal strength is affected by both the interruption of the signal in the time domain and the variation of carriers in the frequency domain. Due to the diverse modulation schemes and medium access control mechanisms, RSSI from many real-life technologies exhibit highly distinctive features. Experimental studies show that these features remain visible when RSSI is computed from samples acquired at a sub-Nyquist sampling rate, making them suitable for narrow-band devices to recognize wideband technologies.

This work investigates two types of features from RSSI, (i) features extracted from the time domain RSSI series (i.e., the distribution of packet duration) and (ii) features acquired from the distribution of RSSI itself. The former can be used for distinction among non-streaming technologies, as illustrated by the case study of Wi-Fi and Bluetooth and discussed in several existing studies; while the latter is a novel contribution of this work, which is verified for application such as detecting the presence of non-streaming technologies and recognizing streaming technologies beyond the ISM bands.

The usage of the RSSI distribution-based feature space is illustrated with a sample algorithm to identify three representative technologies (i.e., Wi-Fi, LTE and DVB-T), which are highly active in the context of dynamic spectrum access. Experimental results show that more than 92% recognition accuracy can be reached under the condition of a 1-MHz ADC sample rate, 5-kHz RSSI collection rate and 50-ms observation time. Literature studies indicate that these conditions are supported by several small-scale and narrow-band devices. Furthermore, considering the operation of LTE-U in ISM bands, the basic feature space is extended for mixed Wi-Fi and LTE signals as a positive first step towards the operation in a heterogeneous wireless environment.

In the context of RSSI-based technology recognition, this work completes existing solutions in the sense that it covers a different set of technologies and uses a different feature space. It should be noted that the focus is not to design a perfect classification algorithm, nor to recognize all technologies exclusively using the RSSI distribution. With this work, we do want to prove that features from the probability distribution of RSSI are complementary to time domain features and remain valid under the sub-Nyquist condition, hence highly valuable to identify wideband technologies on constrained devices, for achieving better dynamic spectrum sharing in the next generation wireless networks.

## Figures and Tables

**Table 1:** The features vs. technologies.

	Tech	Wi-Fi	LTE	DVB-T
Features				
$\sigma_{\vec{R}}$		Variable	High	Low
$N_{p}$		Variable	High	Low
$p(\bar{n})$		High	Not visible	Not present

**Table 2:** Confusion matrix of the measurement results, for *N* = 20, *T* = 1 s.

	Prediction	Wi-Fi	LTE	DVB-T	Noise	Unknown
Actual						
Wi-Fi		92.6%	1.85 %	0%	3.70%	1.85%
LTE		0%	100%	0%	0%	0%
DVB-T		0%	1.85%	98.15%	0%	0%
Noise		0%	0%	0%	0%	0%
Unknown		0%	0%	0%	0%	0%

**Table 3:** Confusion matrix of the extended validation for *N* = 20, *T* = 1 s; noise and unknown signal classes are omitted because of all being 0% results.

	Predicted	Wi-Fi	LTE	DVB-T	Mixed LTE Wi-Fi
Actual					
Wi-Fi		94.4%	0 %	0%	5.60%
LTE		0%	100%	0%	0%
DVB-T		0%	1.85%	98.15%	0%
Mixed LTE Wi-Fi		0%	1.85%	0%	98.15%
